# Beta-gamma phase-amplitude coupling of scalp electroencephalography during walking preparation in Parkinson’s disease differs depending on the freezing of gait

**DOI:** 10.3389/fnhum.2024.1495272

**Published:** 2024-11-13

**Authors:** Yuki Kimoto, Naoki Tani, Takuto Emura, Takahiro Matsuhashi, Takuto Yamamoto, Yuya Fujita, Satoru Oshino, Koichi Hosomi, Hui Ming Khoo, Shimpei Miura, Takahiro Fujinaga, Takufumi Yanagisawa, Haruhiko Kishima

**Affiliations:** ^1^Department of Neurosurgery, Osaka University Graduate School of Medicine, Osaka, Japan; ^2^Institute for Advanced Co-Creation Studies, Osaka University, Osaka, Japan

**Keywords:** phase-amplitude coupling, Parkinson’s disease, freezing of gait, gait disturbance, power spectral density, movement disorder, scalp electroencephalography

## Abstract

**Introduction:**

Despite using beta oscillations within the subthalamic nucleus as a biomarker of akinesia or rigidity in Parkinson’s disease, a specific biomarker for freezing of gait (FOG) remains unclear. Recently, scalp phase-amplitude coupling (PAC) measured through scalp electroencephalography (EEG) has emerged as a promising tool for analyzing brain function. In this study, we examined whether PAC could be a biomarker for FOG.

**Methods:**

We enrolled 11 patients with Parkinson’s disease and recorded scalp EEG in preparation for and during gait while simultaneously assessing motor function, including FOG. We investigated changes in cortical PAC during walking with and without FOG and examined its correlation with the postural instability and gait difficulty (PIGD) score.

**Results:**

Patient characteristics were as follows: mean age 59.1 ± 6.9 years, disease duration 13.9 ± 4.1 years, and seven men. Four trials were excluded from the analysis owing to artifacts. In the trials without FOG (*n* = 18), beta-gamma PAC in the sensorimotor area decreased during gait preparation (*p* = 0.011; linear mixed-effects model), which was not the case in trials with FOG (*n* = 6) (*p* = 0.64; linear mixed-effects model). Using a support vector machine, machine learning of PAC during preparation for walking predicted the presence of FOG with an accuracy of 71.2%. Conversely, PAC increased during walking in trials with FOG (*p* = 0.0042; linear mixed-effects model), and PAC 20 s after the start of walking was positively correlated with the PIGD score (correlation coefficient = 0.406, *p* = 0.032; Pearson’s rank correlation).

**Conclusion:**

Beta-gamma PAC in the sensorimotor area during preparation for walking differs depending on the emergence of FOG. As gait symptoms worsened, beta-gamma PAC in the sensorimotor area during walking gradually increased. Cortical PAC may be a biomarker for FOG in Parkinson’s disease and may lead to the development of strategies to prevent falls in the future.

## 1 Introduction

Akinesia and rigidity in Parkinson’s disease have been linked to increased beta oscillations in the local field potential (LFP) of the subthalamic nucleus (STN) (Kuhn et al., 2006; Kuhn et al., 2009; Weinberger et al., 2009; Neumann et al., 2016b; Neumann et al., 2016a; Neumann et al., 2017; Ray et al., 2008). These findings can provide clues to elucidate the pathology of akinesia and rigidity and serve as biomarkers for deep brain stimulation (DBS) using a closed-loop system. This system is a new strategy that automatically controls DBS based on the STN LFP signal to match the fluctuating motor symptoms of Parkinson’s disease. This new system, recently named adaptive DBS, uses the peak value of beta-band activity in the LFP as a biomarker of motor symptoms, allowing precise adjustments in stimulus intensity to optimize Parkinson’s disease treatment.

Freezing of gait (FOG), a debilitating symptom of Parkinson’s disease, severely impacts patients’ activities of daily living by increasing fall risk and reducing mobility. Unlike akinesia and rigidity, no definitive biomarkers for FOG have been identified. However, recent studies have progressively elucidated the connection between motor symptoms and brain networks in patients with Parkinson’s disease, shedding light on the underlying mechanisms of FOG (Pozzi et al., 2019; Yin et al., 2022).

It has been reported that in the basal ganglia of patients with Parkinson’s disease, there are abnormal neuronal firings, known as beta bursts, with the frequency and duration of these bursts correlating with motor symptoms (Tinkhauser et al., 2017a; Tinkhauser et al., 2017b; Torrecillos et al., 2018; Anderson et al., 2020). In the cortex, gamma-power activity increases during motor, language, visual, and cognitive tasks and is proportional to the increase in the blood oxygen level-dependent signal obtained using functional magnetic resonance imaging (Scheeringa et al., 2011). Electrophysiological and histological evidence suggests that cortical activity is regulated by beta rhythms derived from beta bursts in the basal ganglia, which are modulated through a hyperdirect pathway connecting the basal ganglia to the cortex (Gradinaru et al., 2009; de Hemptinne et al., 2013; Cole et al., 2017; West et al., 2018).

Pozzi et al. (2019) conducted scalp electroencephalography (EEG) on patients with Parkinson’s disease and obtained LFPs from the STN while analyzing the data before and after FOG. They found that the cross power spectral density (PSD) of the STN and cortical theta and alpha bands differed in the presence and absence of FOG, suggesting disruptions in the STN and cortical networks during FOG. This disruption of feedback from the STN due to network failure can cause an increase in beta-gamma phase-amplitude coupling (PAC) in the sensorimotor cortex, resulting in gait disturbance (Pozzi et al., 2019; de Hemptinne et al., 2013).

It has been reported that FOG is affected by visual and cognitive functions in addition to locomotion, suggesting the involvement of impaired networks in the supplementary motor area, which is responsible for motor planning, preliminary movements, and task responses (Pozzi et al., 2019). PAC in the sensorimotor cortex of patients with Parkinson’s disease may reflect not only simple motor function but also projections from other cortical areas, such as those involved in cognition and vision (de Hemptinne et al., 2013). The abnormalities in the beta-gamma PAC of the sensorimotor cortex may reflect a pathological condition that prevents normal responses to information input from other motor control areas, such as the prefrontal cortex or supplementary motor cortex (de Hemptinne et al., 2013). Hence, the PAC from the sensorimotor cortex may contain information on FOG.

We hypothesized that FOG is affected not only by motor function but also by environmental factors, including cognitive and visual information, with physiological changes potentially occurring prior to walking. Thus, we continuously monitored PAC from the preparatory phase of walking, positing that changes in PAC within the sensorimotor area occur at the moment FOG manifests and during the preparatory phase. Identification of biomarkers for FOG in Parkinson’s disease will contribute to the elucidation of the pathology of FOG and has potential applications in fall prevention and closed-loop DBS. This study identified potential biomarkers of FOG in patients with Parkinson’s disease during walking.

## 2 Materials and methods

Scalp EEG was recorded using a portable device (Polimate Pocket MP208, Miyuki Giken, Tokyo, Japan) while patients with Parkinson’s disease were walking. Measurements were taken at random times in both the “on” and “off” levodopa medication states, independent of the patient’s motor status. Gait was captured on a time-stamped video, and the time of gait onset was matched to the EEG time. Motor function was assessed using the Movement Disorder Society-Sponsored Revision of the Unified Parkinson’s Disease Rating Scale (MDS-UPDRS) Part 3 prior to each walking session and was included in the analysis.

### 2.1 Patients and procedure

Between November 2020 and June 2022, of 36 patients with advanced Parkinson’s disease, who had undergone or were scheduled to undergo the implantation of DBS electrodes in the STN at Osaka University Hospital, 11 patients agreed to participate in this study. The patients were diagnosed with Parkinson’s disease based on the Movement Disorder Society Clinical Diagnostic Criteria for Parkinson’s Disease and treated with levodopa. The study protocol was approved by the Ethics Committee of Osaka University (No. 21119), and the study was conducted in accordance with the Declaration of Helsinki. Written informed consent was obtained from the patients, and their rights to voluntarism and withdrawal were ensured.

The surgery was performed in one stage under general anesthesia, with electrodes (DBS lead 3,389, Medtronic Inc., Minneapolis, MN, USA) placed stereotactically on both sides of the STN. An implantable pulse generator (PERCEPT PC, Medtronic Inc., Minneapolis, MN, USA), a closed-loop DBS system only available in Japan, was implanted subcutaneously, mainly in the right chest. A wireless scalp EEG device (Polimate Pocket MP208, Miyuki Giken, Tokyo, Japan) was used for EEG measurements at a sampling rate of 1,000 Hz. EEG was measured using active electrodes placed at the F3, F4, Fz, C3, C4, and Cz positions according to the International 10–20 system. A reference electrode was attached to the midline of the forehead, and electrodes for obtaining extracranial signals were attached to both mastoid processes. The impedance was maintained below 20 kΩ.

The EEG was measured while the participants sat with their eyes open, voluntarily stood up without cues, walked 10 m, made a U-turn, and sat down again. The timing of the start and end of the gait was determined using video (frame rate, 30 beats/s) recorded simultaneously with the EEG. The start of walking was defined as the time when the patient took the first step, and the end of walking was defined as the time when the patient sat down on the chair. For patients implanted with the DBS system, measurements were acquired during stimulation.

### 2.2 Evaluation of clinical symptoms

The MDS-UPDRS Part 3 and Berg Balance Scale were used to evaluate the motor symptoms of Parkinson’s disease prior to the start of walking. Two specialists evaluated these symptoms and FOG. The postural instability and gait difficulty (PIGD) scores were also obtained by extracting the scores of five items from the MDS-UPDRS (2.12, 2.13, 3.10, 3.11, and 3.12) related to gait and posture (Jankovic et al., 1990; Stebbins et al., 2013). Trials were divided into two groups: FOG+ and FOG−. FOG+ was defined as the occurrence of at least one episode of FOG either during the MDS-UPDRS evaluation before walking or during EEG measurement while walking. Conversely, FOG− was defined as the absence of FOG at any time point. This definition was chosen because FOG may not consistently manifest as anticipated; however, this approach allows for the assessment of a brain state that is susceptible to FOG episodes.

### 2.3 Analysis

We analyzed the EEG signals using Python 3.97 with several libraries, including tensorpac 0.6.5, mne 0.24.1, numpy 1.21.4, pandas 1.3.4, and matplotlib 3.5.0. The PAC was calculated using the Tensorpac toolbox^1^ in Python. In patients undergoing DBS stimulation, the recorded EEG signals were notch filtered to attenuate the stimulation frequency. The power supply noise was removed using independent component analysis. Trials with a mean cross-correlation coefficient ≥ 0.8 between the C3 and C4 electrodes and non-cranial signals from the bilateral mastoid electrodes were excluded from this analysis.

The EEG data were separated into 10-s windows. The signal was bandpass filtered into a low-frequency band (10–40 Hz in a 0.5-Hz step, without overlap) and a high-frequency band (50–130 Hz in a 2-Hz step, without overlap) using a two-way finite impulse response filter. A Hilbert transform was performed to convert the low-frequency signal into phase and the high-frequency signal into amplitude.

**Figure 1:**
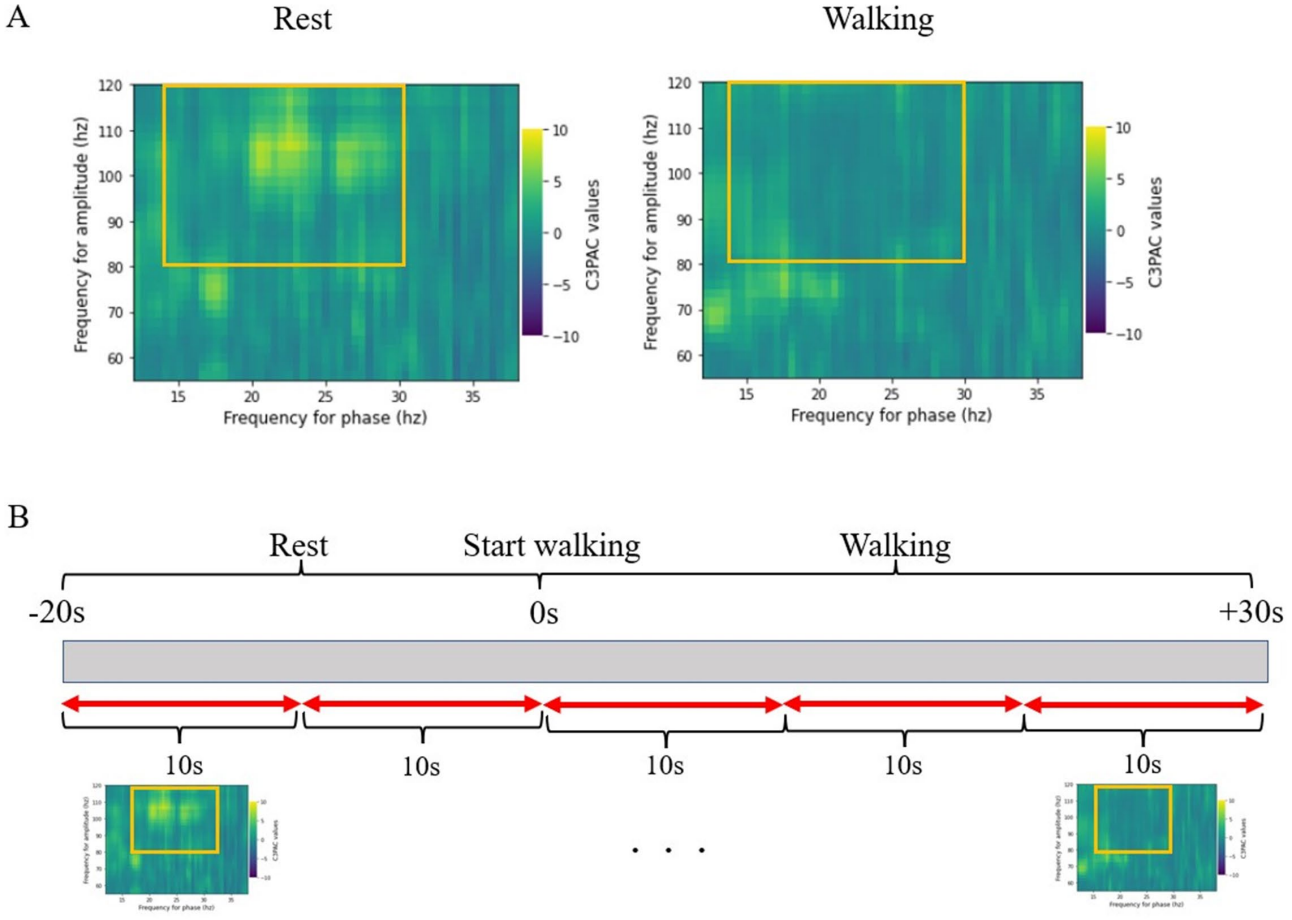
**(A)** Co-modulogram of beta-gamma phase-amplitude coupling. The MI of PAC is standardized into z-scores. The z-scores are averaged over the range of 13–30 Hz for the beta band and 80–120 Hz for the gamma band. **(B)** The period from 20 s before the start of walking to 30 s after the start of walking is divided into 10-s windows, and the MI is calculated for each period for statistical comparisons. EEG, electroencephalography; MI, modulation index; PAC, phase-amplitude coupling.

The PAC value was calculated using the modulation index (MI) (Tort et al., 2010). The MI was derived by calculating the Kullback–Leibler distance that measures the deviation between the probability distribution of the high-frequency amplitude and the uniform distribution. To ensure that the calculated raw PAC did not include signals obtained by chance in the measurements, standardization was performed on the surrogate data using the time-lag method (Canolty et al., 2006). The MI was normalized as a z-score by comparing 200 surrogate data items obtained by dividing the MI by random time with the raw data. The resulting co-modulogram of the MI z-score was averaged within a phase range of 13–30 Hz and an amplitude range of 80–120 Hz (Figure 1A) (de Hemptinne et al., 2013). The MI obtained from this calculation was defined as the beta-gamma PAC in this study.

The period from 20 s before the start of walking to 30 s after the start of walking was divided into 10-s windows, and the MI was calculated for each period for statistical comparisons (Figure 1B). Furthermore, the MI was calculated continuously within the 10-s window with a 0.2-s step size, and we plotted continuous changes in the MI from before the start of walking (resting in a sitting position) to the end of walking. We used a 10-s window in this study because previous reports have shown that this is sufficiently reliable for obtaining the phase from a beta-band signal (Yin et al., 2022). We calculated the correlation between the MI obtained 20 s after the start of walking and the MDS-UPDRS Part 3, PIGD, and Berg Balance Scale scores.

We used Python 3.97’s scikit-learn (version 1.0.1) to analyze the prediction of the presence of FOG from the MI during the walking preparation period (from −20 s to 0 s). This “prediction” refers to whether there is a predisposition to developing FOG rather than whether FOG will actually occur.

Support vector machine (SVM) models were trained with 80% of the dataset, using group k-fold cross-validation (*K* = 10) to predict FOG. The remaining 20% of the dataset (20 s of the MI) was used as test data. Group k-fold cross-validation was performed to prevent the MI from the same patient being divided into training and test data. For support vector machine calculations, we standardized the data and optimized hyperparameters using grid search.

We also investigated the relationship between the beta power from scalp EEG and FOG. PSD calculations were performed in the same window as the PAC calculations using the Welch method. EEG signals were lowpass filtered at 150 Hz, and the PSD was standardized as a z-score for all powers from 1–150 Hz and averaged over 13–30 Hz.

### 2.4 Statistical analysis

When multiple trials were performed for the same patient under the same conditions, weighting was performed to reduce bias. A linear mixed-effects model was used to compare the MI and PSD before and after walking in each group with and without FOG. Channels with large outliers were excluded from the PSD analysis using the Smirnov–Grubbs test.

In this study, we used MI instead of raw PAC values. This was a good method for normalizing raw PAC values, which can vary widely between patients, but because they are relative values, they are not suitable for comparison between groups, so we mainly used a mixed effects model to compare over time. In addition, we did not directly compare the differences in values between groups but instead used a mixed effects model to compare.

We used *t*-tests to compare the patients’ ages, disease durations, Mini-Mental State Examination scores, and Frontal Assessment Battery scores. The Mann–Whitney U test was used to compare the MDS-UPDRS Part 3, Berg Balance Scale, and PIGD scores. The PIGD score and MI 20 s after the start of walking were analyzed using Pearson’s rank correlation. The significance level was set at *p* < 0.05.

All analyses were performed using the R software (R Core Team, 2020, Vienna, Austria).

## 3 Results

A total of 28 EEG measurements (datasets) during walking were performed on 11 patients. The patient characteristics are presented in Table 1. There were no differences in ages, disease durations, Mini-Mental State Examination scores, and Frontal Assessment Battery scores between patients with or without FOG (*p* = 0.51, 0.16, 0.22, and 0.67, respectively; *t*-test). The measurements were performed once or twice under the same conditions for each patient (Table 2). The MDS-UPDRS and Berg Balance scores were evaluated once per patient, with once or twice measurements performed under these conditions. The conditions and patient statuses for each trial are presented in Table 2.

**Table 1:** Patient characteristics.

ID	Age	Sex	Disease duration (years)	MMSE score	FAB score
1	61	M	9	22	10
2	56	F	16	30	17
3	68	M	20	30	N/A
4	60	M	16	30	N/A
5	63	M	9	29	15
6	63	F	17	N/A	N/A
7	63	M	17	29	16
8	45	M	10	29	18
9	52	F	8	30	16
10	53	M	15	30	18
11	66	F	16	30	16

DBS, deep brain stimulation; F, female; FAB, Frontal Assessment Battery; M, male; MMSE, Mini-Mental State Examination; N/A, not available; STN, subthalamic nucleus.

**Table 2:** Conditions and patient status for each trial.

ID	Trial	Dataset	LEDD	DBS (Hz)	UPDRS Part 3 score	Berg balance scale score	PIGD score	Freezing	Inclusion in analysis (yes/no)
1	1	2	675	130	23	47	5	−	yes
	2	2	675	130	25	49	5	−	yes
2	3	1	799.5	145	25	54	5	−	yes
	4	1	799.5	145	24	54	6	+	yes
3	5	1	1137.4	80	42	22	7	−	yes
4	6	2	1,090	130	28	53	10	+	yes
	7	1	1,040	130	21	50	7	+	yes
5	8	1	829	130	21	55	2	−	yes
	9	1	829	130	28	53	3	−	yes
6	10	1	337.5	130	27	53	4	−	yes
	11	1	337.5	130	28	54	4	−	yes
7	12	1	700	60	28	53	8	−	yes
8	13	2	777.5	130	27	56	1	−	yes
	14	2	740	145	6	56	0	−	yes
	15	2	777.5	130	28	56	4	−	no
	16	2	740	145	28	56	1	−	yes
9	17	2	980	N/A	64	51	6	+	no
10	18	2	900	N/A	26	54	6	−	yes
11	19	1	469.7	90	29	51	12	+	yes

DBS, deep brain stimulation; LEDD, levodopa equivalent daily dose; PIGD, postural instability and gait difficulty; N/A, not available; UPDRS, Unified Parkinson’s Disease Rating Scale.

**Figure 2:**
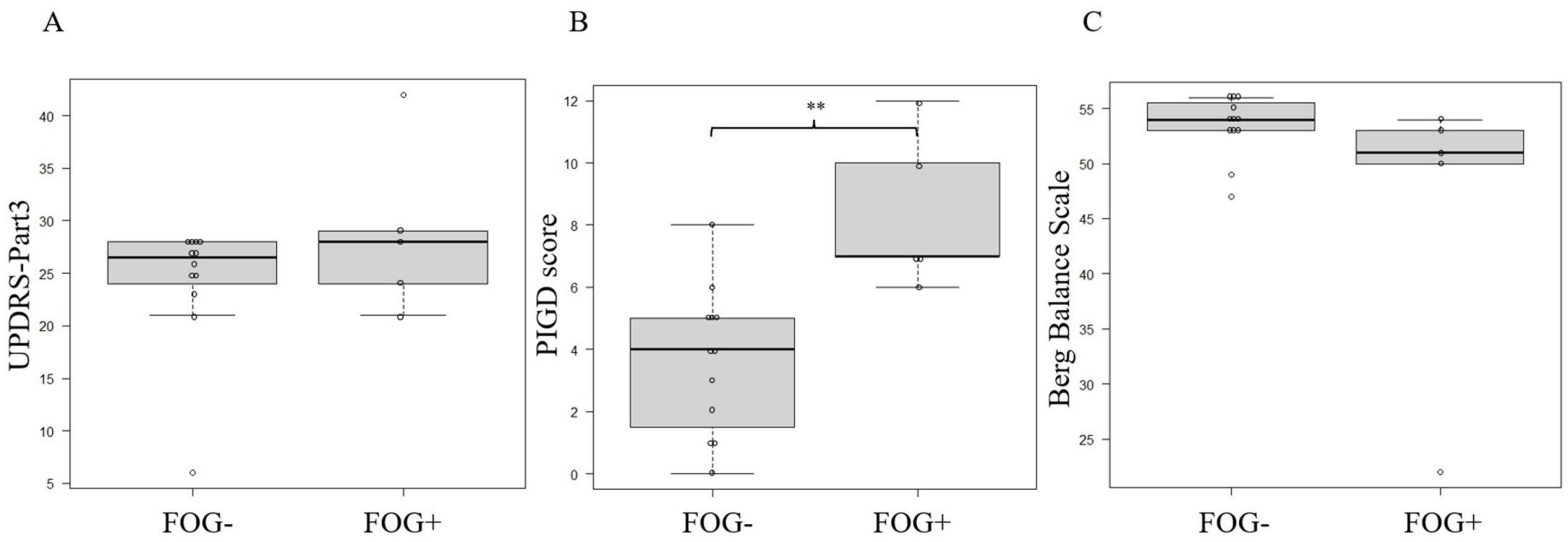
Comparing the MDS-UPDRS **(A)**, PIGD **(B)**, and Berg Balance Scale **(C)** scores between FOG- and FOG+ patients. Asterisks indicate significant differences (**, *p* < 0.01). All error bars displayed represent the range. FOG, freezing of gait; MDS-UPDRS, Movement Disorder Society-Sponsored Revision of the Unified Parkinson’s Disease Rating Scale; PIGD, postural instability and gait difficulty.

The MI obtained from the same patient and under the same conditions was averaged to match the timing of the start of walking to avoid bias across patients or conditions. Data from two trials were eliminated because of artifacts; therefore, 17 trials (24 data sets due to multiple EEG measurements in one trial) were finally analyzed (Table 2), totaling 48 EEG data sets from both C3 and C4 electrodes. There were 36 FOG- EEG and 12 FOG+ EEG data sets. The PIGD score was significantly higher during FOG+ than during FOG-, but there were no differences in MDS-UPDRS Part 3 and Berg Balance Scale (*p* = 0.39 for the MDS-UPDRS Part 3 score, *p* = 0.0059 for the PIGD score, and *p* = 0.097 for the Berg Balance Scale score; Mann–Whitney U test; Figures 2A–C).

**Figure 3:**
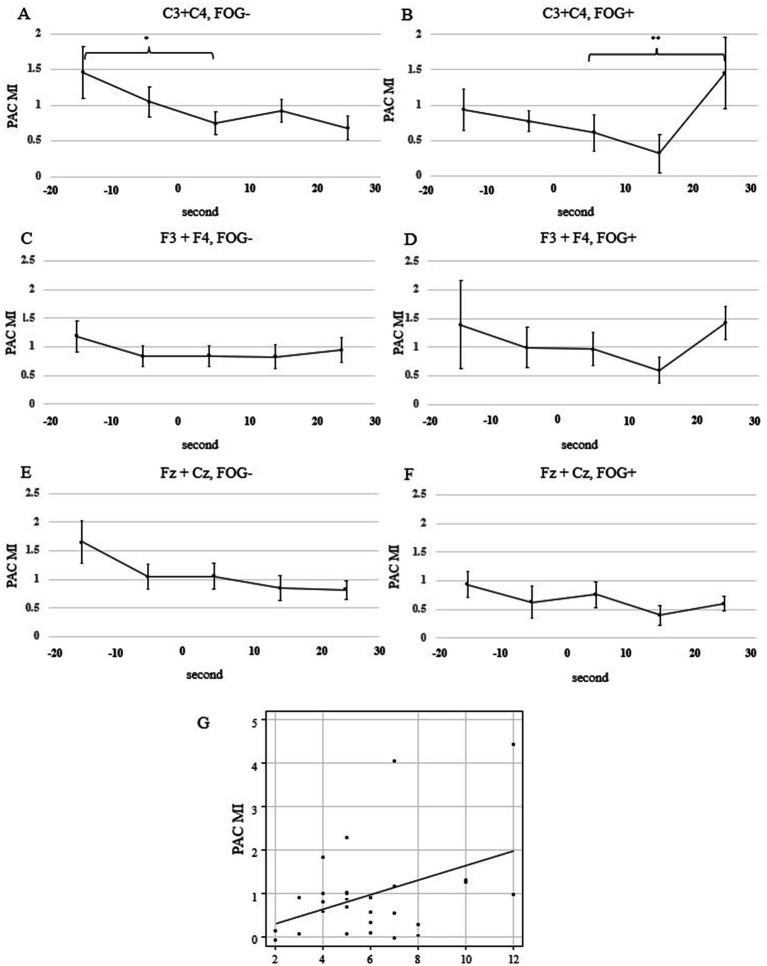
The transition of the mean beta-gamma PAC MI calculated in each 10-s window. **(A)** FOG- in C3 and C4, **(B)** FOG+ in C3 and C4, **(C)** FOG- in F3 and F4, **(D)** FOG+ in F3 and F4 **(E)** FOG– in Fz and Cz, **(A)** FOG+ in Fz and Cz Statistical analysis was performed using the mixed-effects model. Asterisks indicate significant differences (*, *p* < 0.05; **, *p* < 0.01). All error bars displayed represent the standard error of the mean (SEM). **(G)** Correlation between beta-gamma PAC for C3 and C4, obtained from a window of 20–30 s after the start of walking, and the PIGD scores. FOG, freezing of gait; MI, modulation index; PAC, phase-amplitude coupling; PIGD, postural instability and gait difficulty.

In the FOG- trials, the mean MI for C3 and C4 was significantly lower at the start of walking than at 20 s before the start of walking (*p* = 0.011; linear mixed-effects model; Figure 3A). In contrast, in the FOG+ trials, there was no significant difference in the mean MI for C3 and C4 between 20 s before the start of walking and at the start of walking (*p* = 0.64; linear mixed-effects model; Figure 3B). Similar analyses for channels F3, F4, Fz, and Cz revealed no significant changes in MI before the start of walking (*p* = 0.21 and 0.077 for F3 + F4 and Fz + Cz in FOG- trials, respectively; *p* = 0.41 and 0.11 for F3 + F4 and Fz and Cz in FOG+ trials, respectively; linear mixed-effects model) (Figures 3C–F).

We performed 10-fold cross-validation and machine learning, using a support vector machine, to predict the presence of FOG using the MI from electrodes C3 and C4. We used continuous MI (a total of 1,390 samples from 48 EEG data) calculated at 0.2-s intervals from 20 s before the start of walking to 10 s before the start of walking. Using this data, the model predicted FOG with 71.2% accuracy.

We found that the MI increased during walking in the FOG+ trials. The linear mixed effects model used to analyze differences in the time series of MI within the groups revealed no difference in FOG- trials when comparing the MI for C3 and C4 between 20 s after the start of walking and at the start of walking (*p* = 0.40; linear mixed-effects model). However, the MI was elevated in the FOG+ trials (*p* = 0.0042; linear mixed-effects model; Figures 3A,B). No significant increase in the MI after the start of walking was observed for other channels (*p* = 0.52 and 0.94 for F3 + F4 and Fz + Cz in FOG- trials, respectively; *p* = 0.12 and 0.52 for F3 + F4 and Fz and Cz in FOG+ trials, respectively; linear mixed-effects model) (Figures 3C–F).

To compare the differences in PAC after the start of walking between patients with and without FOG, a group comparison was conducted using a linear mixed model for MI during the first 20 s of walking, revealing a significant difference (*p* = 0.0053) between the FOG+ and FOG- groups.

**Figure 4:**
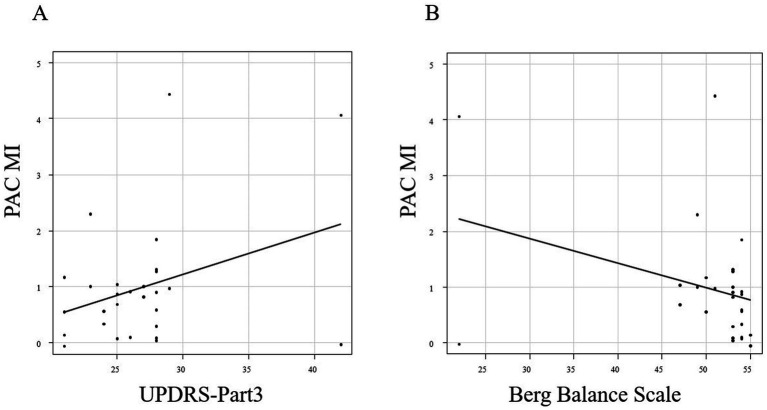
Correlation between the MI 20 s after the start of walking and the MDS-UPDRS Part 3 **(A)** and Berg Balance Scale scores **(B)**.

There was a positive correlation between the mean MI for C3 and C4 at 20 s after the start of walking and the PIGD score, with a correlation coefficient of 0.406 (*p* = 0.032; Pearson’s rank correlation; Figure 3G). The MDS-UPDRS Part 3 and Berg Balance Scale scores showed no correlation with the mean MI for C3 and C4 20 s after the start of walking (*p* = 0.073 and 0.081; Pearson’s rank correlation; Figure 4).

**Figure 5:**
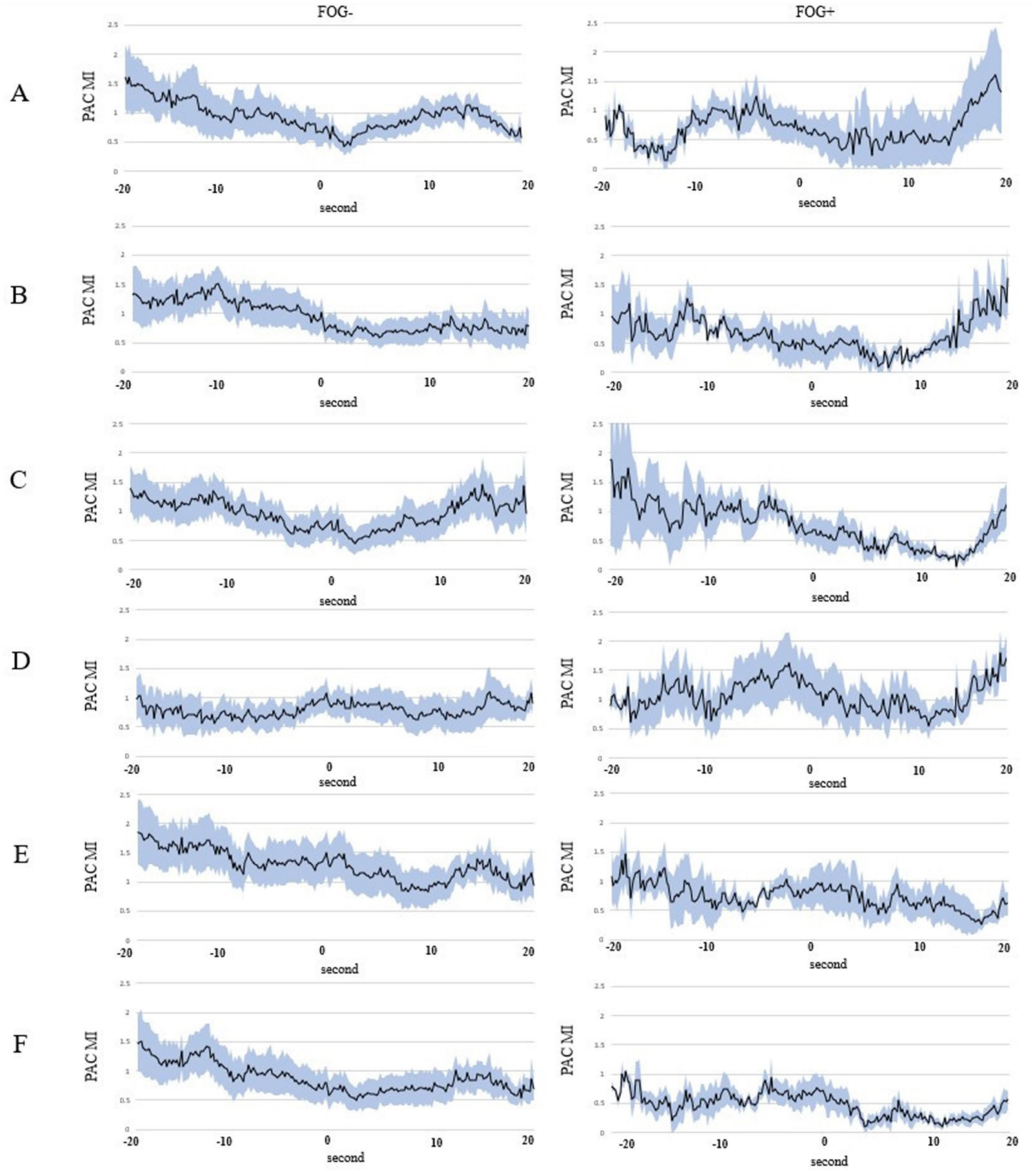
Continuous mean MI plotted at the first point of each window. The blue range indicates the standard error of the mean (SEM). **(A)** C3, **(B)** C4, **(C)** F3, **(D)** F4, **(E)** Fz, and **(F)** Cz. FOG, freezing of gait; MI, modulation index.

The mean and standard error of the MI for each channel, plotted from 20 s before to 20 s after the start of walking, are shown in Figure 5. For C3 and C4, the MI decreased before and remained low after the start of walking in the FOG- trials. Conversely, the MI did not decrease significantly before but increased after the start of walking in the FOG+ trials.

**Figure 6:**
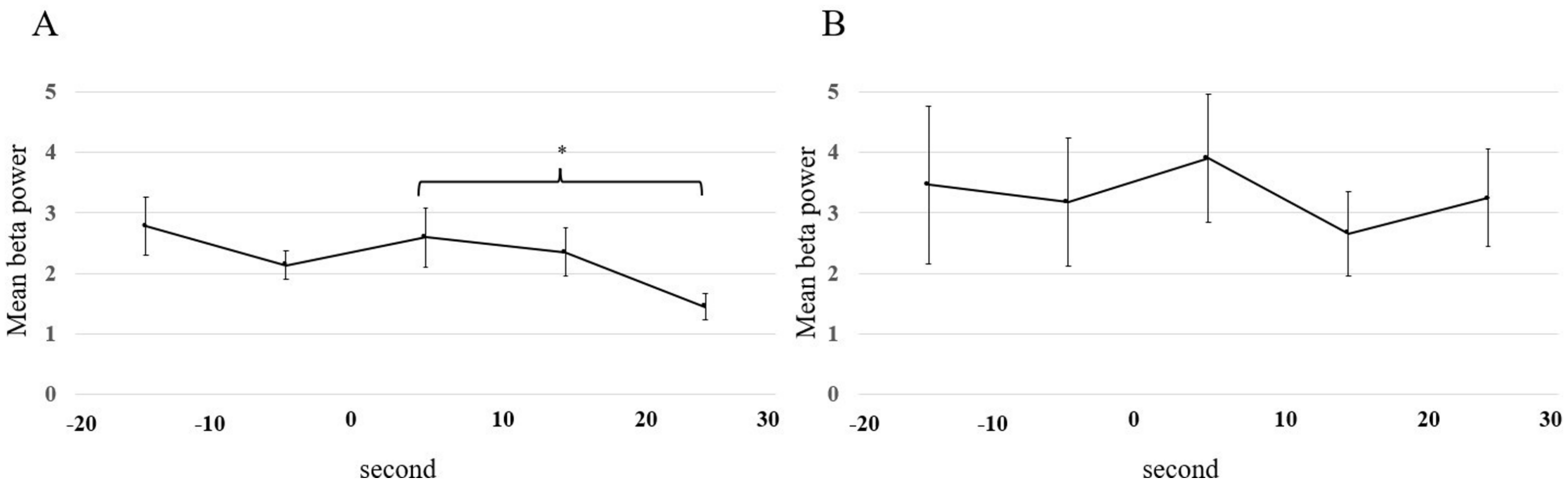
Mean beta power plotted for each 10-s window from 20 s before the start of walking to 30 s after the start of walking. All error bars displayed represent the standard error of the mean (SEM). Statistical analysis was performed using the mixed-effects model. Asterisks indicate significant differences (*, *p* < 0.05). **(A)** FOG- patients. **(B)** FOG+ patients. FOG, freezing of gait.

The mean beta power for C3 and C4 is shown in Figures 6A,B. There was no significant difference in the beta power from C3 and C4 between 20 s before the start of walking and at the start of walking (*p* = 0.78 in FOG-, *p* = 0.881 in FOG+; linear mixed-effects model). After the start of walking, there was a significant decrease in FOG- (*p* = 0.016 in FOG-, *p* = 0.58 in FOG+; linear mixed-effects model).

## 4 Discussion

This study is the first to continuously analyze scalp EEG during walking or walking preparation in patients with Parkinson’s disease, identifying the characteristics of the EEG signal and gait disorders in this patient population. Scalp EEG confirmed that in patients with Parkinson’s disease, beta-gamma PAC in the sensorimotor area decreased before the start of walking in the FOG- group but did not decrease in the FOG+ group. Predisposition to FOG was predicted with an accuracy of 71.2% from PAC data during walking preparation. When walking with FOG, beta-gamma PAC in the sensorimotor area increased after the start of walking, distinguishing FOG+ from the FOG- group. Furthermore, the z-score of beta-gamma PAC at 20 s after the start of walking was positively correlated with the PIGD score.

Yin et al. (2022) used intracranial epidural electrodes to measure the cortical EEG of patients with Parkinson’s disease during walking and found differences in beta-gamma PAC between the resting and walking states. Beta-gamma PAC was reported to be lower during walking than at rest and higher during walking with FOG than during walking without FOG. Our results were consistent with these findings, and we further demonstrated that the difference in beta-gamma PAC between FOG+ and FOG- groups begins from the preparation time before the start of walking. We found that beta-gamma PAC in the sensorimotor area gradually decreased during the walking preparation period in the FOG- trials, and we could predict the emergence of FOG by continuously observing the changes in PAC using sliding 10-s windows in the scalp EEG data.

### 4.1 Phase-amplitude coupling

Beta-gamma PAC in patients with Parkinson’s disease is thought to reflect how pathological beta band signals from the basal ganglia modulate the activity of the gamma band in the cortex, leading to a locked state of cortical activity (de Hemptinne et al., 2013). FOG has been associated with not only motor impairments but also activities involving cortical connections, such as attention, vision, and cognition (de Hemptinne et al., 2013), suggesting a relationship between beta-gamma PAC and FOG.

In this study, the PAC time series analysis revealed that beta-gamma PAC in the sensorimotor area decreased during the pre-walking period in FOG- trials but remained unchanged in FOG+ trials. This finding reinforces the idea that cortical PAC reflects both motor function and preparatory motor activity received from other cortical areas. In addition, we found that PAC did not increase after walking began in the FOG- trial, whereas it did increase in the FOG+ trial, and differences were also observed in the comparison between groups. Consistent with previous reports, it is possible that PAC changes dynamically while walking, potentially contributing to the onset of FOG.

The results of this study provided preliminary data that the PAC of the sensorimotor cortex can be a biomarker of FOG in patients with Parkinson’s disease.

### 4.2 Power spectral density

Many reports have suggested a correlation between the beta power of the LFP of the STN and motor symptoms in Parkinson’s disease (Kuhn et al., 2006; Kuhn et al., 2009; Weinberger et al., 2009; Neumann et al., 2016b; Neumann et al., 2016a; Neumann et al., 2017; Ray et al., 2008). However, the association between the beta power in cortical EEG and motor symptoms remains controversial. In this study, we measured the PSD from scalp EEG during walking and found no significant difference in beta power before the start of walking. Beta power is thought to represent beta bursts (Anderson et al., 2020), a pathological neuronal firing pattern observed in Parkinson’s disease. However, as the sensorimotor cortex is influenced by inputs not only from the basal ganglia but also from other areas of the brain, such as the prefrontal cortex and supplementary motor area, the beta power observed in the cortex is likely to contain information beyond beta bursts from the basal ganglia and may not correlate with motor symptoms. After the start of walking, beta power may decrease with motor performance in the STN (Kuhn et al., 2004). However, in FOG+ states, information from the basal ganglia cannot be reflected in the cortex because of an abnormal connection between the basal ganglia and cortex. Therefore, compared to PAC, we hypothesize that the beta power obtained from the cortex is less likely to show a significant difference between the presence and absence of FOG.

### 4.3 Limitations

This study has some limitations. First, the sample size is small. The patients enrolled in this study were those being evaluated for the potential use of DBS, meaning that most were at a similar stage of Parkinson’s disease, which limited the number of patients. To address this, we performed multiple trials and measurements using multiple EEG channels to increase the sample size.

Second, because scalp EEG was measured during walking, the data may contain signals other than those in the brain. A previous study that examined artifacts of gait using active electrodes found that low-frequency bands (theta, delta, and alpha waves below 12 Hz) were significantly affected by gait, whereas higher frequency bands (beta and gamma waves above 13 Hz) were less affected (Kline et al., 2015). Some artifacts, other than those derived from walking, may have been included even after trials with many artifacts were excluded from the study. However, even under such conditions, there were significant differences in our data based on gait severity.

Third, the evaluation of symptoms in patients with Parkinson’s disease, such as FOG and other gait symptoms, is subjective. There is no established objective evaluation method for Parkinson’s disease as typified by the MDS-UPDRS, and no method can quantitatively measure gait symptoms. Therefore, there is a possibility of some variation in the data. However, in this study, two specialists evaluated FOG and other gait symptoms to minimize this variation, and the interobserver agreement was high.

Finally, we included patients with Parkinson’s disease both before and after DBS treatment, but excluded patients who could not walk at all, limiting our ability to obtain EEG data from patients in the off-medication state who were immobile. The limited variation in the MDS-UPDRS scores, as all patients were able to walk, may explain why no significant correlation was observed between PAC and the MDS-UPDRS Part 3 score. In addition, the absence of data from healthy individuals makes it difficult to determine whether the observations are specific to Parkinson’s disease.

### 4.4 Future clinical and neuroscience contributions

We believe that the findings of this study will help elucidate the pathophysiology of Parkinson’s disease and gait symptoms as part of the disorder of the network connecting the basal ganglia and cortex. A method to measure the biomarkers of gait symptoms may be incorporated into the closed-loop DBS system, which currently only targets akinesia and rigidity. Future analyses of the basal ganglia-cortical PAC relationship will provide further insights into their respective regulatory and feedback relationships. We also measured the PAC of the sensorimotor cortex. Further studies on the coupling between the sensorimotor cortex and other cortical areas can help clarify the brain network involved in FOG and postural dysreflexia. This approach may pave the way for new treatment strategies targeting these network disruptions.

In addition, this finding may be applicable to future brain-computer interfaces (BCI). By combining EEG data with artificial intelligence (AI), BCI can restore motor and sensory functions in paralyzed patients, and attempts have already been made to restore motor function in real time by sending signals to the muscles of quadriplegic patients (Mofatteh, 2021). In addition, by using AI to analyze the vast amount of data obtained from EEG, it may be possible to verify the results of this study and obtain further insights into brain networks.

Although FOG in Parkinson’s disease cannot be treated with current closed-loop DBS system, it is a problem that greatly affects the patient’s activities of daily living. If the occurrence of FOG could be predicted in real time and therapeutic stimulation could be performed on the spot, it may lead to further treatment, including preventing patients from falling.

## 5 Conclusion

In summary, our research demonstrates that beta-gamma PAC in the sensorimotor area, which diminishes during gait preparation without FOG, remains unchanged during gait preparation when FOG is present. Additionally, we observed that as gait symptoms worsen, there is a gradual increase in beta-gamma PAC in the sensorimotor area during gait. This suggests that beta-gamma PAC in the sensorimotor area could be a biomarker for FOG in Parkinson’s disease and may help elucidate the brain network responsible for FOG.

Furthermore, these results suggest that applying BCI to predict FOG while walking may be possible in the future, allowing for the development of real-time intervention strategies to prevent falls.

## Data Availability

The raw data supporting the conclusions of this article will be made available by the authors, without undue reservation.

## Footnotes

^1^https://github.com/EtienneCmb/tensorpac
